# HIV-1 Reverse Transcriptase Inhibition by Major Compounds in a Kenyan Multi-Herbal Composition (CareVid™): In Vitro and In Silico Contrast

**DOI:** 10.3390/ph14101009

**Published:** 2021-09-30

**Authors:** Winnie Rotich, Nicholas J. Sadgrove, Eduard Mas-Claret, Guillermo F. Padilla-González, Anastasia Guantai, Moses K. Langat

**Affiliations:** 1 Sigowet-Soin Sub-County Hospital, Sondu-Kapsoit Road, Sigowet, Kericho County, P.O. Box 112, Kericho 20200, Kenya; rotichwinnie890@gmail.com; 2 Royal Botanic Gardens, Kew, Kew Green, Richmond, Surrey TW9 3AE, UK; E.mas-claret@kew.org (E.M.-C.); f.padilla@kew.org (G.F.P.-G.); m.langat@kew.org (M.K.L.); 3 Department of Pharmacology and Pharmacognosy, School of Pharmacy, University of Nairobi, P.O. Box 19676-00202, Nairobi 00100, Kenya; anguantai@yahoo.com

**Keywords:** CareVid, reverse transcription, in silico, in vitro, chemistry

## Abstract

CareVid is a multi-herbal product used in southwest Kenya as an immune booster and health tonic and has been anecdotally described as improving the condition of HIV-positive patients. The product is made up of roots, barks and whole plant of 14 African medicinal plants: *Acacia nilotica* (L.) Willd. ex Delile (currently, *Vachelia nilotica* (L.) P.J.H Hurter & Mabb.), *Adenia gummifera* (Harv.) Harms, *Anthocleista grandiflora* Gilg, *Asparagus africanus* Lam., *Bersama abyssinica* Fresen., *Clematis hirsuta* Guill. & Perr., *Croton macrostachyus* Hochst. ex Delile, *Clutia robusta* Pax (accepted as *Clutia kilimandscharica* Engl.), *Dovyalis abyssinica* (A. Rich.) Warb, *Ekebergia capensis* Sparm., *Periploca linearifolia* Quart.-Dill. & A. Rich., *Plantago palmata* Hook.f., *Prunus africana* Hook.f. Kalkman and *Rhamnus prinoides* L’Her. The objective of this study was to determine the major chemical constituents of CareVid solvent extracts and screen them for in vitro and in silico activity against the HIV-1 reverse transcriptase enzyme. To achieve this, CareVid was separately extracted using CH_2_Cl_2_, MeOH, 80% EtOH in H_2_O, cold H_2_O, hot H_2_O and acidified H_2_O (pH 1.5–3.5). The extracts were analysed using HPLC–MS equipped with UV diode array detection. HIV-1 reverse transcriptase inhibition was performed in vitro and compared to in silico HIV-1 reverse transcriptase inhibition, with the latter carried out using MOE software, placing the docking on the hydrophobic pocket in the subdomain of p66, the NNRTI pocket. The MeOH and 80% EtOH extracts showed strong in vitro HIV-1 reverse transcriptase inhibition, with an EC_50_ of 7 μg·mL^−1^. The major components were identified as sucrose, citric acid, ellagic acid, catechin 3-hexoside, epicatechin 3-hexoside, procyanidin B, hesperetin *O*-rutinoside, pellitorine, mangiferin, isomangiferin, 4-*O*-coumaroulquinic acid, ellagic acid, ellagic acid *O*-pentoside, crotepoxide, oleuropein, magnoflorine, tremulacin and an isomer of dammarane tetrol. Ellagic acid and procyanidin B inhibited the HIV-1 reverse transcription process at 15 and 3.2 µg/mL^−1^, respectively. Docking studies did not agree with in vitro results because the best scoring ligand was crotepoxide (ΔG = −8.55 kcal/mol), followed by magnoflorine (ΔG = −8.39 kcal/mol). This study showed that CareVid has contrasting in vitro and in silico activity against HIV-1 reverse transcriptase. However, the strongest in vitro inhibitors were ellagic acid and procyanidin B.

## 1. Introduction

In the developing world, a large fraction of the population is dependent on herbal remedies as their primary form of healthcare. In Africa, wild harvested medicines are commonly used in the form of bark or root [1] because it is believed that these organs have a higher accumulation of secondary metabolites. Bark and root medicines are dried, powdered and extracted into an aqueous solution with heat and consumed as a potent tea. While it is common for these therapies to be administered for acute conditions, such as gastrointestinal disturbance or fevers, there is no dearth of clients with chronic diseases, such as human immunodeficiency virus (HIV), that benefit from herbal prescriptions by attenuating symptoms, delaying their inevitable mortality, or treating comorbidities. Although there is plenty of scope for multi-herbal compositions to stand in as adjuvant therapies to mainstream treatments for HIV, the financial barrier to acquiring antiretroviral therapies in the remote regions of Kenya [2] means that such therapies become primary, which has persuaded herbalists to innovate multi-herbal compositions to give people with a lower socioeconomic status who are living with HIV a better quality of life.

As of 2021, the global prevalence of people living with HIV is over 37.7 million [3], with a normal mean of 680,000 people dying annually because of compromised access to life-saving treatments that should ideally be cheap and easily available. HIV has two types—type 1 (HIV-1) and type 2 (HIV-2). HIV-1 is the principal variant that causes chronic disease because it decreases the body’s immune cell count (CD4 cells), leading to acquired immunodeficiency syndrome (AIDS), which predisposes one to life-threatening opportunistic infections [4]. Because of socioeconomic barriers to antiretroviral therapy in African countries, and the suboptimal adherence to these therapies in middle class youth [5], people commonly progress from HIV infection to AIDS. Hence, the control of infections due to HIV is a major challenge in these low-income countries [6,7,8,9].

Numerous reports describe the use of herbal concoctions against HIV and related complications [10,11]. Concomitant with increasing prevalence of HIV infection is the commercialisation of herbal medicines, which has dramatically risen in the last 10 years, as evidenced by the rising number of African traditional medicine (ATM) traders [12]. As part of this upsurge, the CareVid multi-herbal formulation was realised, which has its origins in southwest Kenya, in Kericho county.

The CareVid therapy is now a protected invention that is a popular adjuvant therapy for HIV in Kenya, whose formula is published in several patents [13,14]. As previously mentioned, the herbal product is steeped in hot water and taken as a tea. Currently, there is mounting anecdotal evidence that its composition supports the body’s resistance to viral infection. People living with HIV who have used CareVid reported increased CD4 cell counts [13,14].

The use of traditional medicines presents probable opportunities to discover workable and socially acceptable HIV treatments for use as adjuvants to antiretroviral therapies. The industry also creates jobs and revenue streams for a substantial number of grassroots-level businesses. However, traditional medicines that are purportedly effective against serious diseases should be subjected to some form of empirical analysis to allay concerns regarding safety, efficacy, and additive benefits [15]. Realisation of a positive therapeutic outcome in vitro is feasible because several natural products from plants [16] and marine organisms [17] have already been reported to have anti-HIV effects.

In this study, the tea was extracted and analysed by HPLC–MS to determine the dominant chemical components; then, pure authentic standards were screened for reverse transcription inhibitory effects. HIV-1 reverse transcriptase (RT) has both RNA-dependent ribonuclease H (RNase H) and DNA polymerase (RDDP) activities that work in tandem to translate viral genomic single-stranded RNA to double-stranded DNA, which is then integrated into the DNA of the infected host cell [18]. RT-associated RDDP and RNase H functions are essential for HIV-1 genome replication. Among HIV-1 RT inhibitors, non-nucleoside reverse transcriptase inhibitors (NNRTI) constitute a prominent class of drugs, which, for almost 20 years, has served as the cornerstone of combination antiretroviral therapy (cART) [19,20]. NNRTI are small molecules that bind to HIV-1 RT at a site distinct from the DNA polymerase active site of the enzyme and block HIV-1 reverse transcription via an allosteric mechanism of action [19]. The conformation of RT for RNA hydrolysis is distinctly different from that for DNA synthesis and reveals a structural cavity, which serves as a target for RT inhibition [21].

The specific objectives of the study included: (i) to describe the major chemical constituents of CareVid that are present in CH_2_Cl_2_, MeOH, 80% EtOH in H_2_O, cold H_2_O, hot H_2_O, and acidified H_2_O (pH 1.5–3.5) using HPLC–MS, (ii) to subject the extracts and the major constituents to in vitro HIV-1 reverse transcriptase inhibition, and (iii) to conduct in silico HIV-1 reverse transcriptase inhibition of the major constituents.

## 2. Results

### 2.1. Chemical Analysis

**Figure 1:**
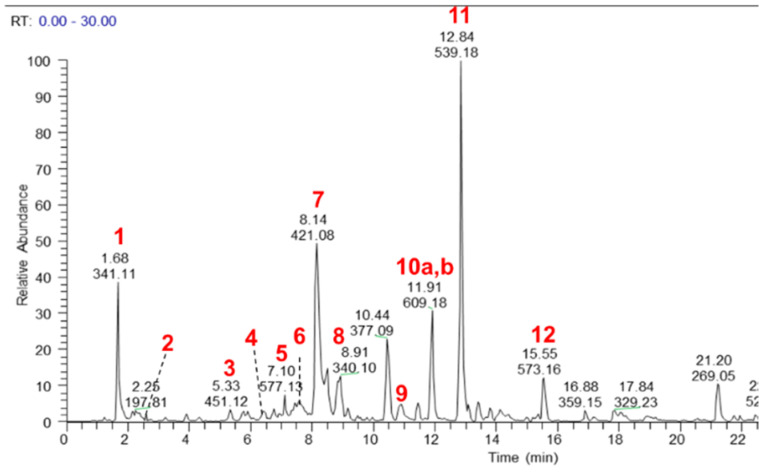
LC–MS base peak chromatogram (negative ionisation mode) of a MeOH extract of CareVid. For compound identities, refer to Section 2.1 or Table S1 in Supplementary Files.

**Figure 2:**
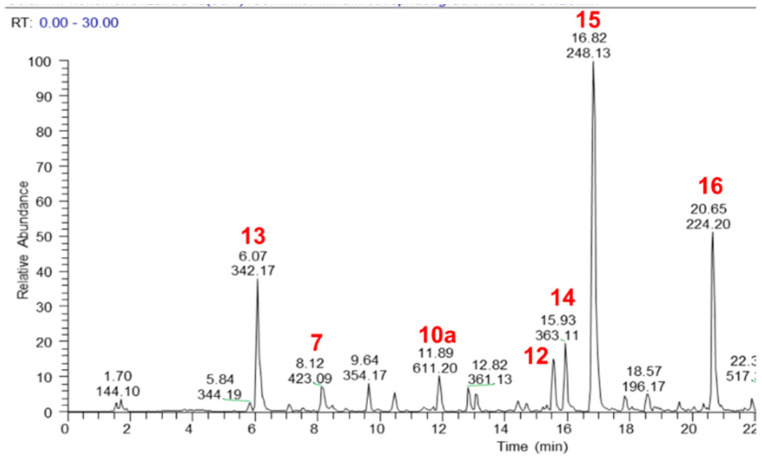
LC–MS base peak chromatogram (positive ionisation mode) of a MeOH extract of CareVid. For compound identities, refer to Section 2.1 or Table S1 in Supplementary Files.

The major components of the MeOH extract were identified as sucrose (**1**), citric acid (**2**), catechin 3-hexoside (**3**), epicatechin 3-hexoside (**4**), procyanidin dimer (**5**), mangiferin (**6**), isomangiferin (**7**), 4-*O*-coumaroulquinic acid (**8**), ellagic acid *O*-pentoside (**9**), hesperetin *O*-rutinoside also known as hesperidin (**10a**), ellagic acid (**10b**), oleuropein (**11**), tremulacin (**12**), magnoflorine (**13**), crotepoxide (**14**), unidentified alkaloid (**15**), pellitorine (**16**) and an isomer of dammarane tetrol (**17**) (Figure 1 and Figure 2, Table S1). Authentic standards were obtained for **5**, **6**, **10a**, **10b**, **11**, **12**, **13**, **14** and **16** and their relative quantities in the MeOH extract was determined semi-quantitatively as 2.8%, 0.54%, 1.41%, 0.49%, 1.83%, 0.92%, 3.26%, 1.8%, and 1.19%, respectively.

Some of these components can be traced to the herbal ingredients by a cross-examination of the published literature, i.e., catechin 3-hexoside [22], ellagic acid and ellagic acid *O*-pentoside [23] have been reported from *Acacia nilotica.* Magnoflorine is expressed by a climber plant on *Acacia nilotica* [24], but is also known from a species in *Croton.* Crotepoxide was reported from *Croton macrostachyus* [25], tremulacin from *Dovyalis abyssinica* [26], and *mangiferin* has been reported from *Bersama abyssinica* [27].

### 2.2. Docking Results

The docking studies were performed using HIV-1 RT in complex with the known inhibitor Nevirapine obtained from the RCSB Protein data bank (PDB ID 1JLB). Nevirapine is a dipyridodiazepinone discovered in 1990 and was FDA approved in 1996 for use as a first-generation NNRTI [28]. Its use in combination with two NRTIs is recommended for first line therapy in developing countries. After the crystal structure of RT complexed with Nevirapine was first published [29], several complexes with RT mutants have been published in order to study the mechanisms of action and drug resistance [30]. Nevirapine, like other NNRTIs, inhibits RT by binding the hydrophobic pocket of the p66 subunit, which has a destabilising effect that reduces the interactions of the polymerase domain with DNA [31].

To validate the docking protocol, Nevirapine was removed from the binding pocket and redocked. The Root Mean Square Deviation (RMSD) value from the known co-crystallised conformation was 0.5582 Å. The method is considered successful when values below 1.5–2 Å are obtained [32]. After method validation, the ligands were energy minimised, and MOE 2015 was used to determine their free energy of binding (ΔG) (Table 1). As expected, the variety of molecular weights and structural motifs of the ligands studied led to a wide distribution of calculated energy of binding values. Crotepoxide **14** and magnoflorine **13** gave the best predicted affinity (ΔG −8.55, −8.39 kcal/mol, respectively), with predicted inhibition constants at the high nanomolar level.

**Figure 3:**
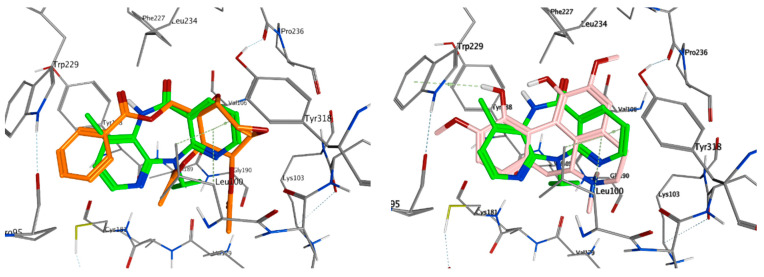
Overlay of top scoring ligands crotepoxide **14** (orange) and magnoflorine **13** (pink) with known inhibitor Nevirapine (green) in the non-nucleoside inhibitor binding pocket portion of reverse transcriptase RT (PDB ID 1JLB).

**Figure 4:**
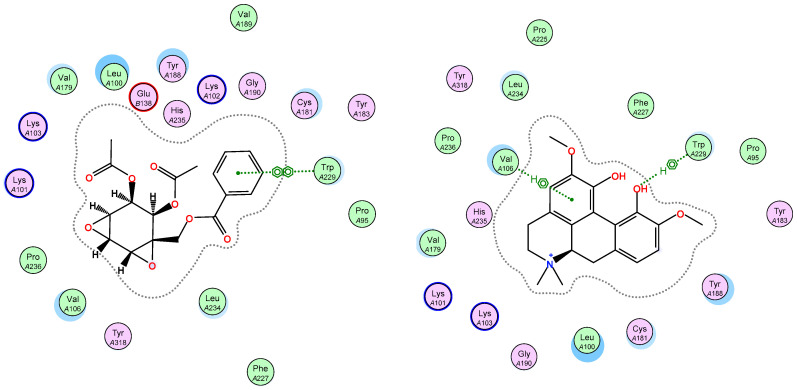
Ligand interactions of top scoring ligands with non-nucleoside inhibitor binding pocket portion of reverse transcriptase RT.

Crotepoxide **14** presents π-stacking interaction between its aryl group and Trp229, as the position of the phenyl moiety shifts slightly compared to that of the pyrido group of Nevirapine (Figure 3 and Figure 4). Presenting an aromatic moiety at this position could contribute importantly to the energy of binding, as it is placed in an aromatic-rich subpocket consisting of Tyr181, Tyr188, Phe227 and Trp229. Trp229 also contributes to the binding affinity of magnoflorine **13**, in this case, with a π–H interaction with one of its hydroxy groups. Additionally, the isopropyl group of Val106 stablishes a second π–H interaction with **13**.

The highest scoring ligand crotepoxide **14** presents one of its acetyl groups occupying a similar position to that of the cyclopropyl group in Nevirapine (Figure 3), thus stablishing a hydrophobic interaction with the pocket found in the active site and contributing favourably to the free energy of binding.

**Figure 5:**
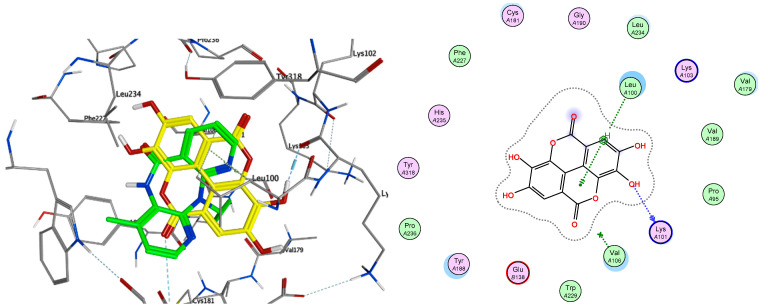
Overlay of ellagic acid **10b** (yellow) with known inhibitor Nevirapine (green) in the non-nucleoside inhibitor binding pocket portion of reverse transcriptase RT (PDB ID 1JLB) (**left**) and ligand interactions of **10b** with non-nucleoside inhibitor binding pocket of RT (**right**).

On the other hand, ellagic acid, which was one of the most active compounds in vitro, showed a slightly higher value of predicted free energy of binding (ΔG −6.80 kcal/mol). In this case, the key π-stacking interactions with aromatic residues in the subpocket are not observed. Instead, there is hydrogen bonding present between Lys101 and one of the hydroxy groups of the ligand (Figure 5). Procyanidin B1 and B2 also showed high in vitro activity, but presented very poor free energy of binding in the docking results, which suggests that their activity could be caused by a different mechanism of action.

### 2.3. In Vitro Reverse Transcription Inhibition

Polar extracts demonstrated the lowest EC_50_ values against reverse transcription using the HIV-1 reverse transcriptase enzyme (Table 2). With values as low as 7 μg·mL^−1^, these alcoholic extracts were slightly more active than the aqueous extracts, which were >10 μg·mL^−1^. Assay of the authentic standards clarified that this activity is related to the presence of procyanidin B and ellagic acid, which had EC_50_ values of 3.2 and 15.3 μg·mL^−1^. Other components that demonstrated moderate activity include tremulacin (539.5 μg·mL^−1^) and hesperidin (624.1 μg·mL^−1^).

## 3. Discussion

In a U.S. Patent (No. 7,556,830), it is described that the CareVid formula was developed after 50 plants known to Kenyan traditional medicine for their anti-infectious properties were assayed to determine potency, and 14 of the most active plants were formulated as CareVid [13,14]. The 14 plants are *Acacia nilotica* (L.) Willd. ex Delile (currently, *Vachelia nilotica* (L.) P.J.H Hurter & Mabb.), *Adenia gummifera* (Harv.) Harms, *Anthocleista grandiflora* Gilg, *Asparagus africanus* Lam., *Bersama abyssinica* Fresen., *Clematis hirsuta* Guill. & Perr., *Croton macrostachyus* Hochst. ex Delile, *Clutia robusta* Pax (accepted as *Clutia kilimandscharica* Engl.), *Dovyalis abyssinica* (A. Rich.) Warb, *Ekebergia capensis* Sparm., *Periploca linearifolia* Quart.-Dill. & A. Rich., *Plantago palmata* Hook.f., *Prunus africana* Hook.f. Kalkman and *Rhamnus prinoides* L’Her (Table 3).

**Table 3:** Documented ethnomedicinal uses of the 14 medicinal plants.

Plant Name	Local Names	Plant Part	Traditional Use on HIV and Other Related Diseases	References
*Acacia nilotica* (L.) Willd. ex Delile	Chebitet	Stem bark	Chest pains, coughs, pneumonia, tuberculosis, coughs Gastrointestinal problems (diarrhoea)	[33]
*Adenia gummifera* (Harv.) Harms	Kinyelwet	Roots	Oral candidiasis, Respiratory infections (chest pains, colds, cough and tuberculosis), Sexually transmitted infections (gonorrhoea and venereal diseases)	[34,35,36,37,38]
*Anthocleista grandiflora* Gilg	Mosombobet	Stem bark	Chest pains	[39]
*Asparagus africanus* Lam.	Birirwapsot	Roots	Tuberculosis, venereal diseases	[40]
*Bersama abyssinica* Fresen.	Cheptorwoget	Stem bark	Sexually transmitted infections (gonorrhoea and venereal diseases)	[41]
*Clematis hirsuta* Guill. & Perr.	Bisingwet	Roots	Pain management Sexually transmitted infections (gonorrhoea and venereal diseases)	[42,43]
*Croton macrostachyus* Hochst. ex Delile	Tebeswuet	Stem bark	Sexually transmitted infections (gonorrhoea and venereal diseases) Chest problems	[44,45]
*Clutia robusta* Pax	Kurbanyat	Roots	Diarrhoea, gonorrhoea, chest pains	[45]
*Dovyalis abyssinica* (A.Rich.) Warb	Nukiat	Roots	Sexually transmitted infections (gonorrhoea and venereal diseases) Gastrointestinal problems	[46,47]
*Ekebergia capensis* Sparm	Arorwet	Stem bark	Respiratory problems (chest pains, cold, cough, respiratory complaints) Sexually transmitted infections (gonorrhoea and venereal diseases) Mouth sores	[48,49]
*Periploca linearifolia* Quart.-Dill. & A.Rich.	Sinendet	Roots	Cough and Pneumonia Sexually transmitted infections (gonorrhoea and venereal diseases), warts, Diarrhoea	[36]
*Plantago palmata* Hook.f.	Chepinderem	Whole plant	Sexually transmitted infections (gonorrhoea and venereal diseases) Gastrointestinal problems	[50]
*Prunus Africana* Hook.f. Kalkman	Tendwet	Stem bark	Prostate cancer, Benign prostate hypertrophy Chest infections Diarrhoea	[51,52]
*Rhamnus prinoides* L’Her.	Kosisitiet	Roots	Sexually transmitted diseases Respiratory infections Gastrointestinal infections Tuberculosis	[53,54,55]

The inventor claims that a female HIV-positive relative who did not respond well to antiretroviral therapy, had her condition improved on using the tea. Subsequently, twenty-six HIV-positive patients in Kericho volunteered to take the decoction, which was administered as a liquid produced by steeping two grams of CareVid in 250 mL of hot water for 5 min, and taken twice a day [13,14]. After two months, two patients felt clinically well and requested a viral load test at a VCT Centre and were determined to be below detection, which is commonly achieved by using antiretroviral therapies but is rarely a validated outcome from exclusive use of natural medicines. By the fourth month six more patients were determined as having viral load below detection. Because none of these patients declared the use of antiretroviral therapies it is of interest if the multi-herbal formulation of CareVid has a stand-alone benefit to human responders in the context of HIV infection [13,14].

While there are various mechanisms by which metabolites can antagonise the life cycle of the HIV-1 virion, the current study focused on a single pathway, the reverse transcription process. For any natural product to achieve therapeutic effects via this pathway, the EC_50_ should conform to a realistic pharmacokinetic metric [56], such as a physiologically relevant concentration and good bioavailability. Hence, ingredients with EC_50_ values at >20 μg·mL^−1^ may be regarded as unrealistic in the translation of in vitro results to in vivo outcomes. Nevertheless, these concentrations were determined as a matter of interest to make a comparison to the results of the in silico study. It was revealed that there was no agreement, and this could be for multiple reasons. The docking approach focused on candidates that are compatible with the binding pocket of nevirapine. Yet, other reverse transcriptase inhibitors, such as zidovudine, antagonise the process by competing with nucleotides and incorporating into the viral DNA strand, causing chain termination due to the lack of a 3′ OH group.

A mechanism for either ellagic acid or procyanidin could be the precipitation of the enzyme, or the compromising of nucleotides. Because of the poor pharmacokinetics of procyanidins [57] and ellagic acid, it may be necessary to consider similar catabolised forms that are created in human digestion. For example, ellagic acid is produced in human digestion by acid hydrolysis of ellagitannins in the stomach. However, ellagic acid is incapable of achieving systemic concentrations high enough to enact the effects of the current in vitro outcome, because high doses of ellagic acid promote microbial processes leading to catabolism of ellagic acid into a urolithin. This creates a high systemic concentration of the urolithins [58]. If urolithins are capable of inhibiting the reverse transcription process at the same concentration as the precursor ellagitannin, then viral inhibition is feasible. It is encouraged to examine the urolithins in a follow up study and ellagic acid may be considered as a suitable prodrug moving forward. Ellagic acid is ubiquitously expressed throughout the plant kingdom, as the hydrolysable precursor of ellagitannin, which is abundant in roots, rhizomes, and bark.

Furthermore, the current study should be followed up by screening against other targets of the HIV-1 virion life cycle, such as integrase or protease inhibition. Nevertheless, if the tea extract is subjected to a bioassay-guided fractionation, then further compounds can be investigated beyond the authentic reference standards used in the current study.

## 4. Materials and Methods

### 4.1. Sample Preparation

Six different types of solvent were used to produce six crude extracts of CareVid Tea powder. Each extract was prepared from 20 g of fresh tea powder. The extract solvents were dichloromethane, ethanol, methanol, water, acidified water (0.1 M HCl), and hot water (>90 °C). These crude extracts were screened in the assay at a starting concentration of 50 μg/mL. The extract that demonstrated the strongest inhibitory activity against the reverse transcriptase enzyme was chemically characterised using HPLC–MS (the methanol extract). The major components that were identified were purchased separately and screened against the reverse transcriptase enzyme in a separate assay.

### 4.2. HPLC–MS Analysis

Metabolic profiling of the methanolic extract was performed by UHPLC–UV–HRMS/MS on a Vanquish UHPLC system (Thermo Scientific, Waltham, MA, USA) coupled to a 100 Hz photodiode array detector (PDA) and an Orbitrap Fusion Tribrid (Thermo Scientific) high-resolution tandem mass spectrometer. Chromatographic separation (5 µL) was performed on a Luna C18 column (150 mm × 3 mm i.d., 3μm, Phenomenex, Torrance, CA, USA) using a mobile phase gradient of 0:90:10 to 90:0:10 (MeOH (A): water (C): acetonitrile + 1% formic acid (D)) over 60 min. Then, 90% A was held for 10 min and returned to initial conditions over 5 min, at 30 °C (flow rate: 400 μL/min). UV detection was recorded between 210 and 550 nm. Mass spectrometry detection was performed in both positive and negative ionisation modes using the full scan and data-dependent MS^2^ and MS^3^ acquisition modes. Total Ion Current (TIC) chromatograms were obtained over the range of 125–1800 *m*/*z* using a spray voltage of +3.5 kV and −2.5 kV for the positive and negative ionisation modes, respectively. Four different scan events were recorded for each ionisation mode as follows: (1) Full scan; (2) MS^2^ of the most intense ion in scan event 1; (3) MS^3^ of the most intense ion in scan event 2; and (4) MS^3^ of the second most intense ion in scan event 2. Additional parameters for the mass spectrometer include: full scan resolution, 60,000 FWHM; capillary temperature, 350 °C; ion transfer tube temperature, 325 °C; RF lens (%), 50; automatic gain control (AGC) target, 4.0 × 10^5^ (Full scan) and 1.0 × 10^4^ (MS^n^); intensity threshold, 1.0 × 10^4^; CID collision energy, 35; activation Q, 0.25; and isolation window (*m*/*z*), 4. Nitrogen was used as the drying, nebuliser and fragmentation gas. The identification of compounds was performed by comparing accurate mass values, UV spectra and MS^2^ and MS^3^ data with literature information and data available in our in-house library of tandem spectra. This library contains more than 5000 tandem mass spectra of isolated or commercially available natural products, including information from some commercially available databases such as NIST. To confirm peak assignments, the fragmentation patterns of each of the identified metabolites were proposed based on MS^2^ and MS^3^ data. Detailed analyses of fragmentation patterns were also used to identify compounds not available in our in-house library. To have an overview of the confidence level achieved in the identification of metabolites, we adopted the four levels of accuracy reported in the Metabolomics Standard Initiative.

### 4.3. HIV-1 Reverse Transcriptase Inhibition Determination

The HIV-1 reverse transcriptase inhibition assay was conducted in vitro using the EnzChek^®^ Reverse transcriptase assay kit (E-22064) provided by Molecular Probes (Eugene, OR, USA), following the manufacturer’s instructions. Reverse transcriptase was purchased from Promega (Madison, WI, USA) (GoScript™ Reverse Transcriptase: catalogue number A5004) and the enzyme dilution buffer from Thermo Fisher (Waltham, MA, USA) (catalogue number B19). Briefly, the EnzChek^®^ RT kit caters to 1000 assays. Assays were performed in increments of 96 using a 96-well microtiter plate. GoScript™ RT was supplied at a concentration of 160 u/μL, but was diluted to 53.3 u/μL in enzyme dilution buffer and frozen as 10 aliquots in volumes of 150 μL at −80°C. A preliminary run was conducted using titrants of the RT to determine the ideal starting concentration, using a ladder with a starting concentration of 26.7 u/μL. A final enzyme concentration of 1.1 u/μL was used, so frozen stock was diluted to 3.4 u/μL in enzyme dilution buffer as the aliquot concentration to give the final concentration of 1.1 u/μL after the reactants were combined. The assay was initiated by annealing of the template and primer. A volume of 5 μL of the poly(A) ribonucleotide template was combined with 5 μL of oligo d(t)_16_ primer and allowed to anneal for an hour at room temperature. After annealing, the 10 μL volume of template/primer was diluted by adding 1.99 mL of polymerisation buffer. This final solution was used as the reaction mixture. The reaction was commenced by combining 20 μL of reaction mixture and 30 μL of treatment, and thereafter adding the 10 μL of enzyme at 3.4 u/μL. The treatment is either the diluted tea extract or pure compound. The reaction was completed after 1 h at 25 °C and terminated with the addition of 30 μL of 15 mM EDTA. Results were determined by adding 180 μL of PicoGreen^®^ dsDNA quantitation reagent solution to the wells and allowing 5 min to pass before reading fluorescence intensity, using 480 nm as excitation wavelength and 520 nm for emission wavelength. The results are reported as concentration of treatment to give 50% enzyme inhibition (EC_50_). Extracts and pure compounds were prepared by dissolving to a concentration of 20 mg/mL in dimethyl sulfoxide and diluting in nuclease free water to double the desired starting concentration. During initial testing, a starting concentration of 50 μg/mL was used, so aliquot concentration was 100 μg/mL to ensure a final concentration of 50 μg/mL. Thereafter, final concentrations of 5–2000 μg/mL were used. All samples and extracts were assayed in triplicate and the averages are provided as results.

### 4.4. Docking Methodology

The docking studies were performed by using the MOE 2015 software. A first energy minimisation of the compounds was performed by MOE using the MMFF94x forcefield, followed by subsequent energy minimisation using Gaussian 09 at the B3LYP/6-31G(d) level of theory. A crystal structure of Y181C mutant HIV-1 RT in complex with Nevirapine was obtained from the RCSB Protein data bank (PDB ID 1JLB). Before docking, the addition of hydrogen and partial charges were performed on 1JLB. To validate the docking protocol, the ligand was redocked into the binding pocket (RMSD 0.5582). Next, the different inhibitor candidates were docked using the triangle matcher method, scoring by London dG, 30 poses, and refined by the rigid receptor, scoring by GBVI/WSA dG, 5 poses.

## Figures and Tables

**Table 1:** Calculated free energy of binding of ligands docked with RT.

Compound Number	Tentative Identity	Free Energy of Binding (ΔG) kcal/mol
**14**	Crotepoxide	−8.55
**13**	Magnoflorine	−8.39
**16**	Pellitorine	−6.94
**10b**	Ellagic acid	−6.80
**11**	Oleuropein	−6.36
**6**	Mangiferin	−4.47
**12**	Tremulacin	−3.74
**5b**	Procyanidin B2	−3.52
**5a**	Procyanidin B1	−2.60

**Table 2:** EC_50_ values for compounds and extracts (treatment group) against the HIV-1 reverse transcription process. A high rating is for values <100 ppm, whereas moderate is 100–1000 ppm. Low activity is above this value. Nil is for no activity detected.

Treatment	EC_50_ ppm	Rating	Treatment	EC_50_ ppm	Rating
DCM	>100	n.a	Catechin	1969.2	Low
H_2_O	15	High	Hesperidin	624.1	Moderate
Hot H_2_O	11.25	High	Hesperitin	4131.7	Low
MeOH	6.9	High	Tremulacin	539.5	Moderate
80% EtOH	7.5	High	Crotepoxide	1368.1	Low
Pellitorine	n.a	Nil	Mangiferin	n.a	Nil
Oleuropein	n.a	Nil	Ellagic acid 1	15.3	High
Magnoflorine	n.a	Nil	Ellagic acid 2	37.2	High
Zidovudine	40.1	High	Procyanidin B	3.2	High

## Data Availability

Data is contained within the article.

## Supplementary Materials

The following are available online at https://www.mdpi.com/article/10.3390/ph14101009/s1, Table S1. Compounds tentatively identified in the MeOH extract from carevid.
