# Supplementary material for: HIV-1 Reverse Transcriptase Inhibition by Major Compounds in a Kenyan Multi-Herbal Composition (CareVid™): In Vitro and In Silico Contrast
Source: Pharmaceuticals (Basel). 2021 Sep 30;14(10):1009. doi: 10.3390/ph14101009 (PMC8541497; doi:10.3390/ph14101009)
Supplement: Supplementary file 1 [file pharmaceuticals-14-01009-s001.zip › pharmaceuticals-1374968-supplementary.pdf]

Table S1. Compounds tentatively identified in the MeOH extract from carevid.

No	Rt (min)	Tentative Identity	Pseudomolecular Ion (*m*/*z*)	MS/MS Ions	Confidence Level
1	1.73	Sucrose	$[M - H]^-$ 341.1086	341 → 281, 179bp, 161, 143, 131, 119, 113, 101	2
2	2.55	Citric acid	$[M - H]^-$ 191.01955	191→ 173, 111bp	2
3	5.33	Catechin *O*-hexoside	$[M - H]^-$ 451.12445	451→ 289bp → 271, 245bp, 231, 205, 179, 125	2
4	6.39	Epicatechin *O*-hexoside	$[M - H]^-$ 451.12424	451→ 289bp → 271, 245bp, 231, 205, 179, 125	2
5	7.10	Procyanidin B	$[M - H]^-$ 577.13464	577→ 559, 451, 425bp, 407, 299, 289, 245	3
6	7.57	Mangiferin isomer 1	$[M - H]^-$ 421.07727	421 → 403, 331, 301bp	2
7	8.14	Mangiferin isomer 2	$[M - H]^-$ 421.07703	421 → 403, 331, 301bp	2
8	8.82	4-O-Coumaroulquinic acid	$[M - H]^-$ 337.09244	337 → 173bp → 155, 137, 127, 111, 93bp, 83, 71	2
9	10.87	Ellagic acid *O*-pentoside	$[M - H]^-$ 433.04092	433 → 301bp → 284, 273, 257bp, 245, 229, 213, 201, 185	2
10a	11.91	Hesperetin *O*-rutinoside	$[M - H]^-$ 609.18188	609 → 301bp → 286bp, 283, 257, 242, 199, 125	2
10b	11.91	Ellagic acid	$[M - H]^-$ 300.99860	301 → 284, 273, 257bp, 245, 229, 213, 201, 185	2
11	12.84	Oleuropein	$[M - H]^-$ 539.17657	539 → 403, 377bp, 345, 307, 275	2
12	15.55	Tremulacin	$[M - H]^-$ 527.15576	527 → 405bp → 377, 299, 219, 155, 137, 123	2
13	6.07	Magnoflorine	$[M + H]^+$ 342.17081	342 → 311, 297bp, 279, 265	2
14	15.93	Crotepoxide	$[M + H]^+$ 363.10822	363 → 321, 303, 261, 243, 215, 199, 181, 153, 139, 121, 105bp	2
15	16.82	Unidentified alkaloid	$[M + H]^+$ 248.12866	248 → 175bp → 145bp	4
16	20.65	Pellitorine	$[M + H]^+$ 224.20135	224 → 168, 151bp, 133, 123, 109, 95, 81, 69	2
17	22.94	Dammarane tetrol	$[M + H]^+$ 443.38910	479→ 461bp, 443 → 425bp, 407, 357, 303	x

Confidence level (Metabolomics Standard Initiative): 1 (high confidence) based upon co-characterization with a reference standard; 2 (medium) based on UV, HRMS and MS/MS comparisons with data in the literature; 3 (low) chemical class suggested by accurate MS and MS/MS; 4 unknown compound; bp: base peak
